# Editorial for Living-donor Lobar Lung Transplantation - Initiation and Development in Japan

**DOI:** 10.31662/jmaj.2024-0024

**Published:** 2024-04-01

**Authors:** Jun Nakajima

**Affiliations:** 1 The Japanese Red Cross Medical Center, Tokyo, Japan

**Keywords:** living-donor lobar lung transplantation, cadaveric lung transplantation, right-to-left inverted transplantation, upper lobe preserving lung transplantation

Professor Date can be said to have dedicated his life to the establishment and development of lung transplantation, especially for living-donor lobar lung transplantation as a clinical practice in Japan ^(1)^. In 1983, Cooper et al. achieved long-term survival after single lung transplantation at the University of Toronto, Canada ^(2)^. Subsequently, lung transplantation from brain-dead donors became common in Europe and the United States. However, in Japan, transplantation from brain-dead donors was not possible until the enactment of the Organ Transplantation Law in 1997.

Additionally, until the requirements for strict and complicated donor organ donation were partly relieved in 2010, the number of organ donations from brain-dead donors in Japan was extremely small. After the amendment of the Organ Transplantation Law in 2010, the number of brain-dead donors increased. By the end of 2023, 971 brain-dead lung transplants and 293 living donor lung transplants had been performed. However, the total number of lung transplants worldwide is less than 1%.

In 1990, lung transplantation using lobe from living donors was performed by Starnes et al at the Southern California School of Medicine ^(3)^. Four cases of living lobe transplantation were reported later, including two cases of bilateral lobe transplantation and two cases of unilateral lobe transplantation (from one donor), with one patient reported to have died early owing to postoperative respiratory failure ^(4)^. It was suggested that obtaining sufficient posttransplant function would require obtaining lower lobes from left and right lung donors.

Compared with lung transplantation using brain-dead donor lungs, in living lung transplantation, donor lungs suffer far less damage and ischemic time is shorter, which favors a successful outcome. Additionally, the surgery date can be scheduled. However, it is necessary to perform surgery on three individuals simultaneously, the lung volume implanted in the donor is smaller, and there is a possibility of insufficient postoperative respiratory function. Moreover, the issue arises of subjecting two healthy individuals to surgical invasion.

As pointed out by Professor Date in his text, compared with brain-dead lung transplantation, the volume of lung lobes after transplantation is obviously smaller, making it a high-risk treatment. In Japan, at the time, finding two suitable donors for patients awaiting the emergence of brain-dead donors was relatively easy. Lung lobe transplantation is technically challenging, and performing it on two healthy donors adds significant surgical stress. However, Professor Date, who bore the greatest responsibility as the surgeon, succeeded in this major performance due to his confidence based on his own skills and knowledge, as well as the advanced teamwork at the Okayama University Hospital in 1998.

The Japanese Society of Lung and Heart-Lung Transplantation publishes a registry report annually, analyzing all lung transplants in Japan. In the status of lung transplantation in 2009, just before the revision of the Organ Transplantation Law, 62 cases of brain-dead lung transplantation and 88 cases of living lung transplantation were observed, a situation much different from that in Western countries where living lung transplantation outnumbered brain-dead lung transplantation. At that time, it can be said that most of the domestic living lung transplants were performed by Professor Date.

Based on numerous cases of living lung lobe transplantation surgeries, new evidence was generated, such as establishing indications for transplant lung lobe volume based on postoperative predicted lung function ^(5)^. Additionally, as mentioned in the main text by Professor Date, inverted lung lobe transplantation, upper-lobe preserving technique, and blood type incompatible transplantation, which are rare with brain-dead donor organ transplantations, were conducted, making it something to be proud of worldwide.

In the field of surgery, especially cardiothoracic surgery, Western countries have been developing advanced surgeries, and Japan has tended to follow suit. In the field of transplantation, owing to social issues, organ transplantation from brain-dead donors has lagged behind in Japan compared with Western countries, and this trend continues to this day. However, within these constraints, the initiation of living lung transplantation in Japan to help patients with diffuse progressive lung diseases and pulmonary hypertension, achieving recipient outcomes comparable with brain-dead donor lung transplantation by Professor Date, ensuring donor safety, and playing a leading role in lung transplantation at many domestic and international hospitals are significant achievements worthy of the Japanese Medical Association Medical Award.

## Article Information

### Conflicts of Interest

None
